# Axenfeld-Rieger Syndrome Associated with Congenital Glaucoma and Cytochrome P4501B1 Gene Mutations

**DOI:** 10.1155/2010/212656

**Published:** 2010-08-09

**Authors:** Mukesh Tanwar, Tanuj Dada, Rima Dada

**Affiliations:** ^1^Laboratory for Molecular Reproduction and Genetics, Department of Anatomy, All India Institute of Medical Sciences, Ansari Nagar, New Delhi 110029, India; ^2^Centre for Ophthalmic Sciences, All India Institute of Medical Sciences, Ansari Nagar, New Delhi 110029, India

## Abstract

Developmental anomalies of the ocular anterior chamber angle may lead to an incomplete development of the structures that form the conventional aqueous outflow pathway. Thus, disorders that present with such dysfunction tend to be associated with glaucoma. Among them, Axenfeld-Rieger (ARS) malformation is a rare clinical entity with an estimated prevalence of one in every 200,000 individuals. The changes in eye morphogenesis in ARS are highly penetrant and are associated with 50% risk of development of glaucoma. Mutations in the cytochrome P4501B1 (*CYP1B1*) gene have been reported to be associated with primary congenital glaucoma and other forms of glaucoma and mutations in pituitary homeobox 2 (*PITX2*) gene have been identified in ARS in various studies. This case was negative for *PITX2* mutations and compound heterozygote for *CYP1B1* mutations. Clinical manifestations of this patient include bilateral elevated intraocular pressure (>40 mmHg) with increased corneal diameter (>14 mm) and corneal opacity. Patient also had iridocorneal adhesions, anteriorly displaced Schwalbe line, anterior insertion of iris, broad nasal bridge and protruding umbilicus. This is the first study from north India reporting *CYP1B1* mutations in Axenfeld-Rieger syndrome with bilateral buphthalmos and early onset glaucoma. Result of this study supports the role of *CYP1B1* as a causative gene in ASD disorders and its role in oculogenesis.

## 1. Introduction

Developmental anomalies of the anterior chamber angle may lead to an incomplete development of the structures that form the conventional aqueous outflow pathway. Thus, disorders that present with such dysfunction tend to be associated with glaucoma. Among them, Axenfeld-Rieger (ARS) malformation is a rare clinical entity with an estimated prevalence of one case in every 200,000 individuals [1]. The term ARS describes a group of congenital diseases that were historically summarized under the broader heading of anterior chamber cleavage syndromes (including Axenfeld anomaly, Rieger anomaly, and Rieger syndrome). ARS represents a spectrum of disorders involving ocular and, in some cases, extraocular structures caused by disruption of migration and differentiation of neural crest cells [2]. Several anterior segment anomalies known to involve tissues derived from neural crest cells have been described in literature and are thought to be due to inadequate regression of tissues and/or failure in differentiation, which results in distortions of normal iridocorneal angle anatomy. These disorders include Axenfeld-Rieger syndrome, Peters anomaly, familial iris hypoplasia associated with glaucoma, and iridogoniodysgenesis. The ocular structures involved in ARS include the cornea (posterior embryotoxon), the iridocorneal angle (peripheral iridocorneal adherences and ultrastructural abnormalities of the trabecular meshwork), and the iris (peripheral adhesions between the iris and the cornea, and stromal atrophy) [3].

Systemic manifestations more commonly associated with ARS include dental abnormalities (microdontia, hypodontia, and oligodontia) and facial malformations (hypoplasia of the maxillary bones) [3]. Redundant periumbilical skin, hypospadias, as well as other less frequent systemic alterations, may also be observed in ARS [4–6]. *Pituitary homeobox 2* (*PITX2*) gene mutations have been identified in ARS in many studies [7, 8]. The changes in eye morphogenesis in ARS are highly penetrant and, anecdotally, have been associated with an approximately 50% risk of the development of glaucoma [9]. Glaucoma may appear during childhood, but it is more common during adolescence or at the beginning of adulthood. Glaucoma secondary to ARS is difficult to manage and may result in severe damage to the optic disc and visual field [3]. In 1997, Stoilov et al., described structural alterations in the *CYP1B1 *gene (MIM 601771), a member of the cytochrome P450 enzyme family, in patients with congenital glaucoma linked to the GLC3A locus [10]. Mutations in the *CYP1B1 *gene have been reported to be associated with other forms of glaucomas, including Peters' anomaly [11–13], and to act as a modifier gene in juvenile open-angle glaucoma [14]. Considering that Peters' anomaly is characterized by anterior ocular malformation because of defective migration of neural crest cells, it is possible to hypothesize that *CYP1B1* gene could play a role in the pathogenesis of ARS.

## 2. Case Details

**Figure 1:**
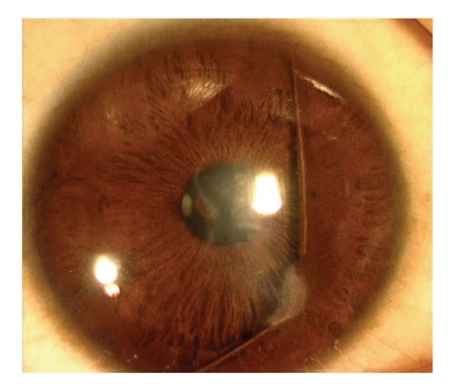
Slit-lamp photograph of eye showing anterior segment dysgenesis.

**Figure 2:**
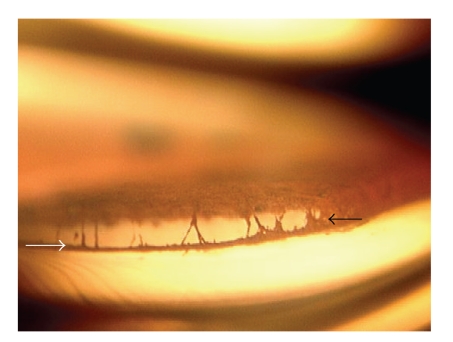
Gonioscopic photograph of eye showing anteriorly displaced Schwalbe line (white arrow) and irido-corneal adhesions (black arrow).

The proband was a 9 months old male offspring who presented with the bilateral buphthalmos with corneal edema. He had intraocular pressure of 48 mmHg and 40 mmHg in the right and left eye, respectively, with corneal diameter of 14.0 × 14.5 mm and 15.0 × 15.0 mm. He had iridocorneal adhesions (Figures 1 and 2), anteriorly displaced Schwalbe line (Figures 1 and 2), and anterior insertion of iris.

On general examination, he had broad nasal bridge and protruding umbilicus. On the basis of ocular and general examination clinical diagnosis of congenital glaucoma with Axenfeld-Rieger syndrome was made. The proband underwent bilateral trabeculotomy + trabeculectomy + mitomycin C (MMC) treatment. Patient is under regular follow up since then. After two years of follow-up he had IOP of 34 mmHg and 20 mmHg in the right and left eye, respectively, with 0.7 : 1 optic nerve cupping in both eyes. Recent corneal diameter was 15.0 × 14.0 mm in both eyes. He also had malaligned teeth.

## 3. Methods

The entire coding region including exon-intron boundaries of *CYP1B1* and* PITX2 *gene from patient and his parents were amplified. PCR for *CYP1B1* gene was done by using three sets of overlapping primers described elsewhere [15]. The primers used were set I (1F-1R, 786 bp), set II (2F-2R, 787 bp), and set III (3F-3R, 885 bp). PCR amplifications for primer sets I and II were performed in a 40 *μ*L volume containing 1.0 *μ*L of 20 *μ*M stock solution for each primer, 100 ng of genomic DNA, 1 unit of Taq polymerase (Banglore Genei), 0.1 mM of each dNTP, 4 *μ*L of 10X PCR buffer (with 15 mM MgCl_2_), and 4 *μ*L of dimethyl sulphoxide (Sigma), by means of 35 cycles of amplification, each consisting of 30 seconds denaturation at 94°C, 30 seconds annealing at 56°C, and 1 minute extension at 72°C [14], while conditions for set III were initial denaturation at 94°C for 3 minutes followed by 30 cycles each at 94°C for 30 seconds, 60°C for 30 seconds, and 72°C for 1 minute and final extension at 72°C for 5 minutes. PCR primers for *PITX2* gene (Table 1) were designed using NCBI PRIMER3 program (available at http://www.ncbi.nlm.nih.gov/tools/primer-blast/). PCR amplifications for *PITX2* all primer sets were performed in a 40 *μ*L volume containing 1.0 *μ*L of 20 *μ*M stock solution for each primer, 100 ng of genomic DNA, 1 unit of Taq polymerase (Banglore Genei), 0.1 mM of each dNTP, and 4 *μ*L of 10X PCR buffer (with 15 mM MgCl_2_), by means of 40 cycles of amplification, each consisting of 30 seconds denaturation at 94°C, 50 seconds annealing at 56–57°C, and 50 seconds extension at 72°C and final extension at 72°C for 5 minutes.

Amplified PCR products were purified using a gel/PCR DNA fragments extraction kit (number DF100; Geneaid Biotech Ltd., Sijhih City, Taiwan). Purified PCR products were sent for sequencing to MCLAB (Molecular Cloning Laboratories, South San Francisco, CA). *CYP1B1 *DNA sequences were analyzed against the *CYP1B1 *gene reference sequence ENSG00000138061 (available at http://www.ensembl.org/Homosapiens/Gene/Sequence?g=ENSG00000138061) and *PITX2* DNA sequences against *PITX2* gene reference sequence ENSG00000164093 (available at http://www.ensembl.org/Homo_sapiens/Gene/Sequence?g=ENSG00000164093) using ClustalW2 (multiple sequence alignment program for DNA; European Molecular Biology Laboratory (EMBL)-European Bioinformatics Institute (EBI).

## 4. Computational Assessment of Missense Mutations

Two homology based programs PolyPhen (Polymorphism Phenotyping), available at http://genetics.bwh.harvard.edu/pph/, and SIFT (Sorting Intolerant From Tolerant) analysis tool, available at http://sift.jcvi.org/ were used to predict the functional impact of missense changes identified in this study. PolyPhen structurally analyzes an amino acid polymorphism and predicts whether that amino acid change is likely to be deleterious to protein function [16–18]. The prediction is based on the position-specific independent counts (PSIC) score derived from multiple sequence alignments of observations. PolyPhen scores of >2.0 indicate the polymorphism is probably damaging to protein function. Scores of 1.5–2.0 are possibly damaging, and scores of <1.5 are likely benign. SIFT is a sequence homology-based tool that sorts intolerant from tolerant amino acid substitutions and predicts whether an amino acid substitution in a protein will have a phenotypic effect [19–22]. SIFT is based on the premise that protein evolution is correlated with protein function. Positions important for function should be conserved in an alignment of the protein family, whereas unimportant positions should appear diverse in an alignment. Positions with normalized probabilities less than 0.05 are predicted to be deleterious and those greater than or equal to 0.05 are predicted to be tolerated.

## 5. Results

*PITX2* gene analysis showed heterozygous T > A change at g.11553261 (in Intron 5). No pathogenic *PITX2* mutation was identified in this patient. *CYP1B1* analysis showed presence of two heterozygous pathogenic mutations (p.Arg355Stop + p.Arg368His). Both of these mutations (*CYP1B1*) have been reported in cases with primary congenital glaucoma [15]. Father of the proband was negative for these mutations but mother was heterozygous for p.R368H mutation.

### 5.1. Arginine355Stop (p.R355X) Mutation

**Figure 3:**
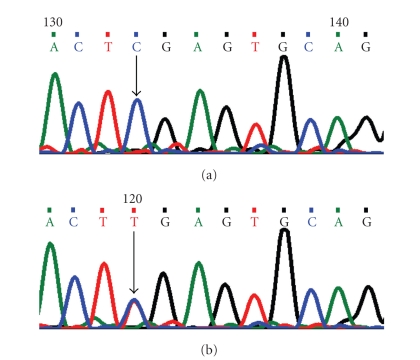
DNA sequence chromatogram of *CYP1B1* exon 3 equivalent to codon 354–357. (a) The reference sequence derived from control is shown. (b) Sequence derived from ARS patient shows heterozygous c.1063C > T, which predicts a codon change CGA > TGA and heterozygous p.R355X, a non-sense mutation.

In this mutation, a single-base cytosine (C) was replaced by thymine (T) (Figure 3) at genomic position g.38151938; coding nucleotide number c.1063. This resulted in a codon change from CGA to TGA and p.R355X a non-sense mutation in *CYP1B1* protein. This resulted in a truncated *CYP1B1* protein of 354 amino acids.

### 5.2. Arginine368Histidine (p.R368H) Mutation

**Figure 4:**
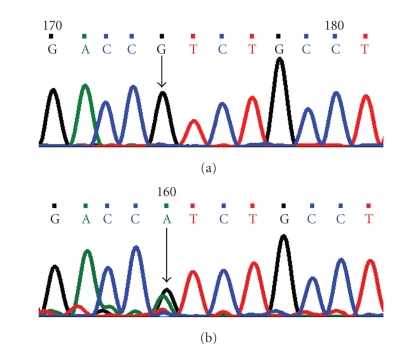
DNA sequence chromatogram of *CYP1B1* exon 3 equivalent to codon 367–370. (a) The reference sequence derived from control is shown. (b) Sequence derived from ARS patient shows heterozygous c.1103G > A, which predicts a codon change from CGT > CAT and heterozygous p.R368H mutation.

A single-base guanine (G) was replaced by adenine (A) at genomic position g.38151898; coding nucleotide number c.1103. This resulted in a codon change from CGT to CAT and amino acid change from arginine to histidine (p.R368H) a nonsynonymous mutation (Figure 4). PSIC score of this mutation was 2.653 indicating that this change is probably damaging to protein function. SIFT score of p.R368H is 0.00 and is predicted to be deleterious for the protein function.

Since, missense mutation in *PITX2* was absent no computational assessment was done for *PITX2* protein.

## 6. Discussion

Over the last several years, the identification of genes and loci involved in the different forms of glaucoma has led to a better understanding of the pathogenetic mechanisms of primary open-angle glaucoma, congenital glaucoma, and developmental glaucoma, such as the one associated with ARS [2]. Although AR has an autosomal dominant inheritance there have been reports of sporadic cases in the literature [23].

Mutations of *CYP1B1* are a major cause of primary congenital glaucoma in various studies from different populations [15, 24–29]. *CYP1B1* is a member of the cytochrome P450 super family of drug metabolizing enzymes. It catalyzes several oxidative reactions, some of which are biosynthetic, producing necessary hormones or compounds of intermediary metabolism in most living organisms [30]. It also metabolizes vitamin A in two steps to all-trans-retinal and all-trans-retinoic acid. The latter is a potent morphogen and regulates in utero development of tissue growth and differentiation [31–33]. It is involved in the metabolism of the endogenous and exogenous substrates that take part in early ocular differentiation [31–33]. This case was compound heterozygote for p.R355X and p.R368H mutations. In p.R355X mutation, a truncated protein of 354 amino acids is produced. The arginine residue at position 355 lies in the carboxyl terminal of the J helix, carboxyl terminal of the J helix is involved in the functionally important heme-binding domain. This change (p.R355X) was first reported in PCG in Germany and after that from India [15, 34]. This truncating mutation results in a loss of the heme-binding domain and a functionally inactive protein [10, 34–36]. Mutations in *CYP1B1* gene emerged as a largest cause of PCG and p.R368H has been reported as most predominant *CYP1B1* mutation in PCG in Indian population [24].

Arginine residue at 368 position lies in between the helices J and K in an exposed loop [24, 29]. Consequences of this change are not immediately apparent except that the positively charged amino acid arginine is replaced by histidine whose charge state depends upon its protonation state. In the WT, arginine at 368 interacts with G-365, D-367, V-363, and D-374. Because of the p.R368H mutation, interactions between D-367 and D-374 are weakened [36]. PolyPhen and SIFT score of p.R368H showed that this mutation is deleterious for the protein function. An alteration of *CYP1B1* expression due to a sequence change might alter the metabolic activity of *CYP1B1*, thus cause ocular development defects. Membrane-bound cytochromes, such as *CYP1B1*, have a molecular structure containing a transmembrane domain located at the N-terminal end of the molecule. This is followed by a proline-rich “hinge” region, which permits flexibility between the membrane-spanning domain and the cytoplasmic portion of the protein molecule. The COOH-terminal ends are highly conserved among different members of the cytochrome P450 super-family [37]. This family contains a set of conserved core structures responsible for the heme-binding region of these molecules. The heme-binding region is essential for the normal function of every P450 molecule. Between the hinge region and the conserved core structure lies a less conserved substrate-binding region. The cytochrome P450 protein functions like any classical enzyme molecule [32, 33]. Mutations affecting such enzymes generally produce recessive phenotypes because in heterozygous subjects the normal allele is capable of compensating for the mutant allele. Mutations in the *CYP1B1* protein interfere with the integrity of the *CYP1B1* protein as well as its ability to adopt a normal conformation and to bind heme; for example, induced mutations in the hinge region have previously been reported to interfere with the heme-binding properties of the cytochrome P450 molecules [38].

First report of mutations in *CYP1B1* as a cause for Rieger Anomaly was published in 2006 by Chavarria-Soley et al. [39]. Three reports exist of *CYP1B1* mutations in Peters Anomaly; patients are either compound heterozygotes [11, 13] or homozygous in consanguineous families [12].

Segregation of both ARS and Peters' anomaly in the same family has been reported previously [40–43]. Therefore, ARS and Peters' anomaly are discussed to be allelic variants and part of the single disease spectrum of anterior segment dysgenesis (ASD) rather than representing distinct entities. This hypothesis is supported by clinical and genetic findings as well as by the embryonic pathogenesis of ASD. First, several clinical features like corectopia or angle anomalies are common both in ARS and in Peters' anomaly. Second, mutations in FKLH7/FOXC1 genes (as well as mutations in other genes implicated in ASD) are associated with clinically variable manifestations like ARS [44, 45], primary congenital glaucoma [24–26], and Peters' anomaly [40]. Third, ASD is genetically heterogeneous.

Peters' anomaly, for instance, has been associated with mutations in PAX6 [42, 46–48], *CYP1B1* [11–13, 49], and FOXC1 [43]. Fourth, there is growing evidence that the ASDs are neural crestopathies that result from a developmental arrest of specific anterior segment tissues derived from neural crest cells [3]. Mutations in *CYP1B1 *could be contributory in up to 20% of cases of Peters anomaly. Since this case was negative for pathogenic *PITX2* mutations, therefore, results of this study support the role of *CYP1B1 *as a causative gene in ASD as suggested by previous studies [11–13, 39, 49]. Furthermore, this emphasizes the broad range of phenotypic expression for *CYP1B1 *mutations, and its role in eye development.

## 7. Conclusion

This is the first study from India reporting *CYP1B1* mutations in Axenfeld-Rieger syndrome with bilateral buphthalmos and early onset glaucoma. Result of this study supports the role of *CYP1B1 *as a causative gene in ASD disorders [11–13, 39, 49] and it emphasizes the broad range of phenotypic expression for *CYP1B1 *mutations, and its role in oculogenesis.

## Figures and Tables

**Table 1:** *PITX2* primers used in this study.

Primer Name	Primer Sequence	Product Size
PITX1F	5′ CAC TCC CGC TGC CAT TGC GT 3′	618 bp
PITX1R	5′ GGG GGC TTC GGT ACA CAG CG 3′	
*PITX2*F	5′ ACC GGG GAG GCG CAG AAA GA 3′	635 bp
*PITX2*R	5′ GCC GAG GTT TGC TGG AGC GT 3′	
PITX3F	5′ GCA GCC CAG CTC TTC CAC GG 3′	559 bp
PITX3R	5′ GTG AGA TCG CGG GAT GGC GG 3′	
PITX4F	5′ CTG CGC TTG GTG GAG ACC CG 3′	812 bp
PITX4R	5′ GTT GCC CCA TCC GGC AAG GT 3′	
